# Thickening agents used for dysphagia management: effect on bioavailability of water, medication and feelings of satiety

**DOI:** 10.1186/1475-2891-12-54

**Published:** 2013-05-01

**Authors:** Julie AY Cichero

**Affiliations:** 1 School of Pharmacy, The University of Queensland, Brisbane, Queensland, Australia

**Keywords:** Thickened liquids, Dysphagia, Gum, Starch, Satiety, Medication, Bioavailability

## Abstract

Dysphagia is the medical term for difficulty swallowing. Thickened liquids are often used in the management of dysphagia to improve bolus control and to help prevent aspiration. A range of starches and gums has historically been used to thicken liquids. Although thickened liquids improve swallow safety, they appear to have a great potential for unintended physiological consequences. Initial concerns were raised about the impact of thickeners on water binding due to the high prevalence of dehydration amongst individuals with dysphagia. Thankfully, regardless of thickening agent, thickeners do not affect water bioavailability. This effect holds true even for extremely thick fluids. However, bioavailability of medication is impaired with viscous substances. Liquids thickened to as little as 150 mPa.s retards drug release. In addition, feelings of satiety and thirst increase with increasingly viscous fluids. Flavour deteriorates with increasing thickness regardless of thickening agent. Therapeutically clinicians often prescribe small volumes of thickened liquids, consumed often. Yet small volumes of thick substances consumed with a long oral processing time, which is common for individuals with dysphagia, reduces the amount consumed. A combination of poor flavour, and increasing feelings of fullness result in little motivation and poor physiologic drive to consume thickened liquids.

This review provides evidence from the dysphagia, pharmaceutical and food technology literature to show unintended side effects of thickened liquids that contribute to dehydration and potential sub-theraputic medication levels for individuals with dysphagia. The physical property of viscosity rather than a particular thickening agent appears to be key. Provision of “spoon-thick” or “extremely thick liquids” is particularly likely to contribute to dehydration and poor bioavailability of solid dose medication. Clinicians are encouraged to prescribe the minimal level of thickness needed for swallowing safety. Consultation with pharmacy and dietetic staff is essential for optimum management of individuals with dysphagia. Given the aged population forecasts for the year 2050, improved dysphagia management should be a high priority.

## Review

The percentage of people aged 65 yrs or more is growing rapidly, causing governments to plan urgently for the long-term implications of aging with one in 13 anticipated to require aged care [1,2]. The National Research Council of the National Academies (USA) is anticipating that the ratio of people aged 65+ yrs to those aged 20–64 years will increase by 80% by the year 2050 [1]. In Australia, people over 70 years are expected to make up 20% of the population by 2051[2]. Better medical care, improved medication and public health promotion has improved life expectancy. Individuals are surviving stroke, and many are living to an age where the specter of dementia becomes more of a certainty. The ability to eat and drink is essential to survival and fundamental to the social fabric of society. Swallowing difficulty (dysphagia) has a prevalence of 13% in community dwelling elders, approximately 25% of hospitalized individuals and rises to 60% or more of elderly residents living in skilled nursing facilities [3-7].

Dysphagia results from many conditions such as: stroke, Motor Neurone disease, Parkinson’s disease, dementia, head and neck cancer, head injury and others. Problems may occur with oral preparation, or containment of food or liquids within the mouth. Difficulty may be experienced coordinating breathing, swallowing and bolus transport in order that the bolus enters the oesophagus rather than the airway. Complications of dysphagia include chest infection, and in some cases death due to choking on food, or as a result of aspiration pneumonia. In order to reduce the likelihood of aspiration, liquid thickness is often altered.

During oral manipulation and swallowing, liquid flow is turbulent, resulting in eddies and vortices [8]. Healthy people can tolerate these factors and cleanly direct liquids past the airway and into the oesophagus. Individuals with dysphagia, however, find the turbulent and fast flow of liquids difficult to control during passage through the pharynx, resulting in impaired airway protection. One of the methods of managing this challenge is to thicken liquids in order that they flow more slowly, allowing the individual time to coordinate safe swallowing [9]. Thickened liquids are not a diet of choice, but one of safety, and have been used therapeutically to manage dysphagia for about 19 years [10]. Although international standards are currently lacking, a review of the recent literature suggests there are at least three levels of viscosity used for the management of dysphagia (Table 1) [11]. Typically the least viscous liquid (akin to the thickness of nectar) is used for mild dysphagia, whilst increasingly thicker liquids are used to manage more severe forms of the condition. Prescription of fluid thickness is patient specific.

**Table 1:** **Three levels of fluid thickness commonly used in dysphagia treatment**[11]**and commercial comparisons**[10,28]

**Least thick**	**Moderate thickness**	**Most thick**
*Labels commonly used*:	*Labels commonly used*:	*Labels commonly used*:
Nectar-thick	Honey-thick	Spoon-thick
Level 150 – Mildly thick	Level 400- Moderately thick	Level 900 – Extremely thick
Stage 1	Stage 2	Stage 3
*Viscosities reported in this range:*	*Viscosities reported in this range:*	*Viscosities reported in this range:*
51-350 mPa.s	351-1750 mPa.s	> 1750 mPa.s
150 mPa.s	400 mPa.s	900 mPa.s
**Comparison thickness with commercially available products**:		
Water	1 mPa.s	
Chocolate milk	56 mPa.s	
Whipping cream	100 mPa.s	
Chilled tomato soup	250 mPa.s	
Tomato sauce	800 mPa.s	
Chocolate custard	1870 mPa.s	
Pureed peaches	2000 mPa.s	

The prevalence of the use of thickened fluids has only been studied comprehensively for the aged care demographic. Of 25,470 residents in a skilled nursing facility, a mean of 8.3% and range of 0-28% of residents received thickened fluids for the treatment of dysphagia [12]. Most patients who required thickened liquids received nectar-thick fluids (30-60%), a smaller percentage received honey-thick fluids (18-33%), whilst only a small proportion received spoon-thick fluids (6-12%) [13].

Liquids are thickened with a range of starches and gums. Starches such as modified corn starch swell, whilst gums cause meshes of entanglement that water molecules become lodged in [14]. Over the past six years there has been a steady migration away from starch towards gum-based thickeners. However, reports have emerged about the safety of gum thickened fluids for infant consumption following the death of a small number of infants due to nectrotising enterocolitis [15]. For adult use, apart from having poor flavour attributes, thickened liquids are generally considered by clinicians to be benign [16]. The emerging literature raises questions as to how thickeners interact with liquids and their effect on a range of important functions. This paper will review the literature for the impact of thickened liquids on hydration, medication bioavailability and physiologic feelings of satiety.

### Water-binding capacity of thickened liquids: implications for dehydration

Liquids are essential for adequate hydration. Water is a necessity for biochemical actions within and outside of cells. Desirable water intake for older adults is calculated at 25–30 ml/kg/day [17]. This equates to between 1.7-2 litres of fluid per day. Dehydration is a common concern for people with dysphagia [18,19]. 75% of individuals in long-term care have been reported to be dehydrated when relying on thickened liquids for oral hydration [20]. Dehydration increases the chances of falls, the risk of renal failure, constipation, urinary tract infection, impaired mental status, respiratory infection, poor muscle strength and ulcers associated with being bed-bound [21]. Starches and gums (Table 2) are used in the food, pharmaceutical and cosmetic industries for their ability to gel and/or bind water. Starch is generally broken down through all phases of digestion. This starts in the mouth with amylase, and then progresses through hydrolytic enzymes in the stomach and further processes in the small intestine where ultimately water and nutrients are absorbed. The gums on the other hand, and particularly the galactomannans, tend to pass through the upper phases of digestion relatively untouched with enzymes in the microflora of the large intestines responsible for their breakdown [22]. Consequently, concern has been raised about the water–binding capacity of gums, and their potential contribution to inadequacies of hydration in individuals with dysphagia.

**Table 2:** Properties of thickening agents used in the treatment of dysphagia

**Thickener**	**Solubility**	**Ionic charge**
Xanthan gum (polysaccharide )	Water soluble	Highly negative charge (anionic)
Guar gum (carbohydrate – galactomannan – manose to galactose ratio 2:1)	Water soluble	Neutral
Locust bean gum (galactomannan – manose to galactose ratio 4:1)	Water soluble	Neutral
Starch (polysaccharide)	Water soluble	Neutral
Carageenan (sulphated linear polysaccharide)	Water soluble	Negative charge (anionic)

There appear to be only two studies that have specifically investigated the bioavailability of water when mixed with thickening agents, used for dysphagia treatment. Sharpe et al. showed in both rat and human studies that liquids thickened to ‘pudding-thick’ level did not affect the bioavailability of water [14]. The studies used blood and saliva sampling. Rats consumed tritiated thickened water and humans consumed deuterium oxide and sodium bromide to label the water. The results showed that water was rapidly absorbed and equilibrated within 60 minutes and that water absorption exceeded 95% of the administered dose. Furthermore, starch (modified maize starch) and gum thickeners (guar and xanthan) were assessed against pure water, with rates of absorption for all test fluids identical. These results demonstrate that thickener type does not affect bioavailability of water. Hill et al. further support these results using a stable isotope methodology and urine sampling in a single subject design [23]. Hill et al. demonstrated that water bioavailability was unaffected by water thickened with xanthan gum to ‘pudding-thick’ level (mean 0.97 ± 0.06 standard error) [23]. Both of these studies conclude that water is made available from solutions containing thickeners commonly used for the treatment of individuals with dysphagia.

### Factors affecting insufficient consumption of thickened liquids

**Figure 1:**
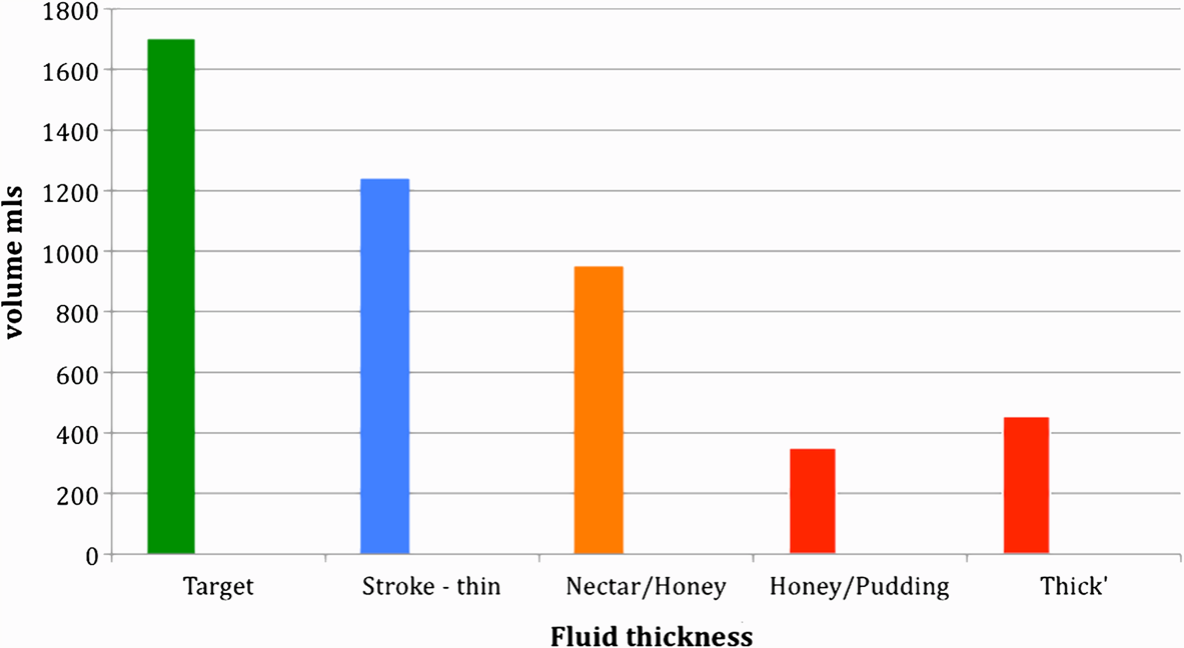
**Daily hydration target (1.7-2.0 litres) **[17 ]**and comparison volume of thin or thickened liquids consumed by individuals with dysphagia.** Legend: ‘Stroke thin’ – amount of un-thickened liquids consumed by stroke patients; ‘Nectar/honey’ – amount of nectar- or honey-thick liquids consumed by individuals with dysphagia; ‘honey/pudding’ – amount of honey- or pudding thick liquids consumed by individuals with dysphagia; ‘thick’ – amount of thickened liquids consumed by individuals with dysphagia of unspecified level of thickness [25,55].

If there is sufficient availability of water from thickened liquids, then other factors must influence the dehydration that is commonly associated with dysphagia. Volume of thickened liquid consumed, thirst quenching ability and flavour of thickened liquids can all affect patient compliance and daily intake. Results consistently demonstrate that individuals who require thickened liquids consume less than if they were to consume un-thickened liquids [24,25] (Figure 1).

Hospitalised individuals have been found to have insufficient access to containers of liquids, difficulty opening drink containers, and difficulty accessing staff to assist them with drinking [26]. Furthermore, increasing levels of thirst have been statistically associated with increased levels of disability. Although Blower’s study related to patients in oncology, orthopaedic and general medical wards, the results are relevant to all hospitalised individuals, including those with dysphagia. Liquid-*access* issues will contribute to reduce daily intake and, are largely remediable. Factors around inability of thickened liquids to quench thirst are more challenging, however. When the mouth is wet, as occurs with an influx of saliva and wetness provided by liquids, oral signals are conveyed to the brain to signal that thirst has been quenched and drinking behaviour can cease. Thirst will persist if the oral phase is bypassed, even if the person is physiologically hydrated by direct infusion of water to the stomach [27]. Anecdotally, individuals with dysphagia complain of thirst and that thickened liquids leave a coating feeling inside the mouth. Interestingly, a study of healthy individuals demonstrated that thirst sensation progressively worsened with increasing viscosity [28].

Not only are thickened liquids poor at quenching thirst, they also result in poor flavour release. A number of studies have demonstrated that once polymers reach the critical point of random coil overlap (c*) and form entangled networks that flavour perception decreases with increasing viscosity [29]. Flavour suppression and ‘off flavours’ of thickened liquids have been reported by Matta et al. [16]. Starch based thickeners were found to impart a starch flavour and a grainy texture for nectar- and honey-thick consistencies. Gum based thickeners did not produce grainy textures, but do produce a higher ‘slickness’ than starch based thickeners. Flavour suppression was demonstrated for all thickening agents. A combination of poor flavour plus poor thirst quenching ability may begin to explain why patients consume less thickened liquids than un-thickened liquids.

### Bioavailability of medication and thickened liquids

Medication has become the main stay of medical treatment. More than 60% of drugs are marketed as oral products, although some may be better suited to alternative delivery methods. Oral ingestion is seen to be: more convenient, have high compliance, safety, efficiency, ease of accessibility and low production costs [30]. An ageing population has an increasing need for medications. Dysphagia affects the ability to safely swallow oral medications, in addition to liquids and foods.

Delivering the right amount of medication to the correct part of the body at the right time is a complex equation. Factors such as preferred absorptive site of the body and solubility and permeability of the solid dose medicine (Table 3) must be considered [31].

**Table 3:** **Biopharmaceutics Classification System of medications **[31]

	**High solubility**	**Low solubility**
**High permeability**	**Class I**	**Class II**
	High solubility	Low solubility
	High permeability	High permeability
	Transporter effects minimal	Efflux (flows out) transporter effects predominate
	*Examples:* Atropine, Caffeine, Diazepam, Glucose, Levodopa, Prednisolone	*Examples:* Digoxin, Ibuprofen, Warfarin
**Low permeability**	**Class III**	**Class IV**
	High solubility	Low solubility
	Low permeability	Low permeability
	Absorptive transporter effects predominate	Absorptive and efflux transporter effects could be important
	*Examples:* Atenolol, Captopril, Cimetidine, Acyclovir, Penicillin, Amoxicillin, Erythromycin	*Examples:* Amphotericin B, Chlothalidone, Neomycin

Medications have preferred absorptive sites to maximise their effect. For example, buccal or sublingual administration is designed to be absorbed through the oral mucosa and its rich blood supply. The stomach has a large epithelial surface, however, contents stay in the stomach for a relatively short period of time giving a short window for absorption. Food delays gastric emptying. Medications that need to be absorbed quickly will be taken on an empty stomach. The small intestines provide for fast absorption and a large surface area. The time taken for medication to transit the small intestines will affect absorption rate and hence bioavailability of the drug. Other medications, such as sustained release doses, are best suited to absorption from the colon. As noted above, guar gum and xanthan gum tend to be broken down by the bacterial microflora of the colon. The literature shows that both of these gums allow for medication release to be delayed until the solid dose reaches the colon because of this feature [32].

Class I and III medications have been implicated for poor bioavailability when combined with thickened liquids. The release of prednisolone (Class I), used in the treatment of inflammatory and auto-immune disorders, could be delayed by increasing concentrations of a mixture of xanthan gum and locust bean gum [33]. In a careful double blind study guar gum was found to have minimal effect on the absorption of digoxin (Class II), a medication used to treat congestive heart failure. However, absorption of penicillin (Class III) was found to be significantly reduced when mixed with guar gum at a concentration of 5gr to 200 ml water [34].

Increasing viscosity impedes drug dissolution and disintegration. A 150 mPa.s viscosity solution (hydroxypropyl methylcellulose [HPMC] as thickener) impeded dissolution of paracetamol such that for all formulations, less than 40% of the drug had been dissolved by 60 mins, although drug solubility was not affected. Film coating adds another dimension and resulted in only 30% of the drug being dissolved after one hour in a viscous solution [35].

Although viscosity impedes drug dissolution and disintegration, a further complication presents in the form of electrical charge associated with the thickener and the way it interacts with the solution. Sarisuta et al. demonstrated that when solutions of similar viscosity were compared for their effect on drug dissolution, that the negative electrical charge on xanthan gum (anionic) impeded dissolution more than the neutral charged (ionic) polymers of guar gum and HPMC [36].

Some of the characteristics of gum thickeners (mucoadhesion) may be beneficial to absorption sites such as the buccal or nasal mucosa. Some drugs are not absorbed well in the intestines or are eliminated too quickly through the gastro-intestinal tract. Buccal, or in some cases nasal drug delivery, is an option for these types of medication. Both bucaal and nasal inserts have been formulated with (xanthan + locust bean gum) and (xanthan + guar gum) respectively [37,38]. The nasal inserts showed excellent bioadehesion and sustained drug release for a medication to reduce nausea and vomiting (metoclopromide hydrochloride) in the treatment of cancer chemotherapy, migraine, pregnancy and gastroparesis [38]. The bioadhesive quality of the gums explains difficulty with mouth wetting and persistent feelings of thirst noted previously.

Many of the studies reported above have been carried out using solid dose forms. The effect of crushing medication and mixing it with thickened liquid for bioavailability is the subject of ongoing research at the University of Queensland (Associate Professor Kathryn Steadman, personal communication).

### Effect of thickened drinks on satiety

Satiety relates to the state of being fed or gratified to, or beyond capacity. Sharpe et al. hypothesised that one of the reasons why insufficient thick liquids are consumed is due to thickened liquids triggering gastric stretch receptors to satiety more so than thin liquids [14]. There are no studies that have looked specifically at the effect of thickened liquids used for individuals with dysphagia and their effect on satiety. However, the obesity and food technology literature provides help in determining the merits of this hypothesis.

Satiety cues have been reported from three different physiological phases: (a) the oral stage (perception of taste and texture) (b) the gastric phase (distension and emptying), and (c) the intestinal phase (distension and absorption) [39]. Human and animal studies agree that increasing the thickness of a liquid reduces the amount consumed when compared with an unthickened liquid [40,41]. A number of confounders have been put forward suggesting that elements other than viscosity can explain increased satiety including: energy density, fibre content, oral processing time, and gastric fullness sensation.

The food literature clearly demonstrates that increasing nutrient and caloric load slows gastric emptying [39,40]. However, in healthy individuals 30-34% more thin liquids were consumed than semi-liquid and semi-solid products when all substances were equal for energy content, energy density, volume and macronutrient composition. There were statistically significant reductions in amount consumed, despite similar subjective feelings of satiety. Progressively less was consumed as fluid thickness increased [28]. The test substances were similar in thickness to slightly thick, moderately thick and extremely thick liquids used in the management of dysphagia (Table 1).

Incorporated air has also been used to increase volume whilst keeping energy density, taste, and macronutrient contents the same [42]. Compared with the control condition, all aerated samples reduced the amount of food consumed immediately following by 12%. The aeration of liquid samples has important implications for the preparation of thickened liquids for people with dysphagia. In aged care skilled nursing facilities and hospitals where thickened liquids are made in bulk, large-scale industrial mixers are often used. Rolls et al. study suggests that aeration must be kept to minimum to reduce feelings of satiety in individuals with dysphagia [42].

The fibre content of thickeners may impact on feelings of satiety. Gums are known in the food industry as a source of dietary fibre, with fibre playing an important role in gut health and digestion. Gums represent the ‘new generation’ of thickeners, having gained popularity over modified starch. Drinks thickened with modified starch have been shown to be unstable, frequently continuing to thicken, or over-thicken, over time [43]. Fibrous foods have been found to reduce hunger and appetite [44]. Liquids with added fibre also appear to provide more feelings of fullness, with guar gum showing the highest satiety ratings [44]. Two elements confound these results, however. Firstly the guar gum beverage in the study was 1000 times thicker than the non-fibre containing control liquid. Secondly, the error bars for each of the samples tested were very wide, indicating large inter-individual variation. Heini et al. found that partially hydrolysed guar gum did not affect mouthfeel, texture or satiety [45]. Slavin et al. reported that liquids containing non-viscous fibre (e.g. inulin) did not have a satiating effect, even at high doses [46]. Hoad concured that it is not fibre, but the physical property of viscosity that increases feelings of satiety [39].

Oral perception of thickened liquids has been implicated as a factor contributing to satiety. In primates, unimodal (viscosity) and multimodal (viscosity + taste) neuronal representation has been found in the orbitofrontol cortex, demonstrating that viscosity is an important perceptual feature [47]. Increased viscosity slows oral transit and affords more time for the oral receptors to be exposed to the taste and texture of thickened liquids. When semi-solids (equivalent in thickness to ‘extremely thick’ liquids) are taken in small amounts and with long oral processing time, healthy individuals consume 1.2-1.3 times *less* than if larger mouthfuls and shorter oral processing time is used [48] (Table 4). For individuals with dysphagia this poses a real problem. Post stroke, individuals may take 10 sec + for oral processing due to tongue weakness and oral incoordination [49,50]. Speech pathologists sometimes recommend teaspoon-sized mouthfuls to reduce aspiration risk [51,52]. The physiological response to mouthful size and oral processing time inherently places individuals with dysphagia who require very thick liquids, at risk of dehydration.

**Table 4:** **Quantity of semi-solid (viscosity 788 mPas) consumed with variation according to weight and time in oral processing**[48]

	**3 sec Oral Processing**	**9 sec Oral processing**
5 gram	382 gr	313 gr
15 gram	476 gr	432 gr
Free bite size = 10 gr ± 4		

Several studies have looked at gastric fullness sensation. During typical food ingestion, what was initially a homogenous mass starts to separate such that there are ‘liquid-like’ and ‘solid-like’ areas. The stomach selectively empties more dilute material whilst continuing to work on the more ‘solid-like’ material. High-resolution colour-coded images acquired through echoplanar magnetic resonance imaging shows that the same process occurs with locust bean gum and alginate thickened substances [39,40]. However, liquids thickened with guar gum did not show phase separation, resulting in a homogenous gastric mass [39]. After passage through the stomach and duodenum, chyme enters the small intestines for nutrient absorption. If nutrient absorption is slow, satiety signals continue to be generated. Hoad et al. (2004) suggests that signals from the small intestines may well have a role with thickened liquids, as satiety signals continue despite gastric emptying [39].

Incredibly, where stomach volume is the same volume, a viscous substance increases feelings of satiety more than a non-viscous substance [40]. The hypothesis put forward here is that sensation from the oral phase, through associative learning, triggers feelings of satiety rather than simply relying on signals from the stomach or small intestines. Indeed McCrickerd et al. have shown that upon seeing drinks of different thickness (ranging in viscosity from 10–317 mPa.s at shear rate 50 sec^-1^) that participants *expected* to feel more full from ingesting the thicker drinks [53]. It has been hypothesised that textural qualities such as thickness provide cues as to the energy load within the drink.

## Conclusion

Due to medical and pharmaceutical advances many people are living well beyond 65 years of age. Data pertaining to world population aging suggest that by the year 2050 one fifth of the population of the developed world will be over the age of 60 yrs and that for the first time in history there will be more elders than young people [54]. Dysphagia prevalence increases with age. Dysphagia disturbs the very foundation skills of eating and drinking. Thickened liquids are used as one form of management for dysphagia. However, this treatment modality is not without complications. This paper has demonstrated that although water is made bioavailable from all thickeners to even extreme levels of thickness, that individuals with dysphagia are frequently dehydrated. The literature on satiety suggests that dehydration may be due to physiological expectations that thick fluids will make them feel full. Flavour suppression associated with increasing thickness provides little motivation to drink. The mucoadhesive qualities of many thickeners leave the mouth feeling sticky after a drink rather than wet, resulting in continuing unresolved feelings of thirst. Clinical recommendations to take small amounts of thickened fluids may result in less being consumed. Neuromuscular impairment that results in delayed oral transit further compounds the effect of reduced intake. Taken together, the literature review suggests that individuals prescribed very thick liquids (e.g. ‘spoon-thick’ or ‘Extremely thick fluids – Level 900’) will struggle to meet hydration needs orally. Individuals receiving moderately thick (e.g. ‘honey-thick’ or ‘moderately thick fluids - Level 400’) will also perceive their drinks to be more filling and will consume less than those on unthickened beverages. The dietitian has an essential role in monitoring and preventing dehydration.

Administration of medication with thickened liquids needs careful consideration and consultation with a pharmacist. Effects of delayed dissolution and disintegration have been demonstrated from as little as 150 mPa.s level viscosity, (just thicker than whipping cream) with increasing adverse effects noted for increasing thickness. Class III BCS drugs appear to be particularly vulnerable. The ionic charge on thickening agents has a cumulatively negative effect on drug release. Given that medication is the main stay of treatment for the elderly to manage stroke, heart conditions, progressive neurological impairment, pain and infection, these initial findings are sobering. In vivo studies of individuals with dysphagia are required to demonstrate the true effects of thickened liquids on medication bioavailability and satiety. Clinicians should strive to prescribe only the least thickened liquid required for swallowing safety and aggressively pursue treatments to improve functional return to normal, un-thickened liquids.

## Abbreviations

HPMC: Hydroxypropyl methylcellulose; mPas: Milli pascal seconds; BCS: Biopharmaceutics Classification System.

## Competing interests

Support for the work reported in the manuscript was provided by Trisco Foods. JC has been an educational speaker for the Nestlé Nutrition Institute and Wyeth.

## Author’s contribution

The author alone was responsible for the content and writing of the manuscript.

## Author’s information

JC (BA, BSpThy [Hons], PhD) is a speech pathologist and deglutitionsit. She holds an honorary senior lecturer position with the School of Pharmacy, and has conducted and published research in the area of thickened liquids with the School of Chemical Engineering, The University of Queensland, Australia since 1996.
